# Time-variant reproductive number of COVID-19 in Seoul, Korea

**DOI:** 10.4178/epih.e2020047

**Published:** 2020-06-28

**Authors:** Seong-Geun Moon, Yeon-Kyung Kim, Woo-Sik Son, Jong-Hoon Kim, Jungsoon Choi, Baeg-Ju Na, Boyoung Park, Bo Youl Choi

**Affiliations:** 1 Department of Preventive Medicine, Hanyang University College of Medicine, Seoul, Korea; 2 Seoul Center for Infectious Disease Control and Prevention, Seoul, Korea; 3 Division of Medical Mathematics, National Institute for Mathematical Sciences, Daejeon, Korea; 4 Epidemiology, Public Health, Implementation & Clinical Development Unit, International Vaccine Institute, Seoul, Korea; 5 Department of Mathematics, Hanyang University College of Natural Sciences, Seoul, Korea; 6 Bureau of Civil Health, Seoul Metropolitan Government, Seoul, Korea

**Keywords:** COVID-19, Communicable disease, Epidemics, Seoul

## Abstract

**OBJECTIVES:**

To estimate time-variant reproductive number (R_t_) of coronavirus disease 19 based on either number of daily confirmed cases or their onset date to monitor effectiveness of quarantine policies.

**METHODS:**

Using number of daily confirmed cases from January 23, 2020 to March 22, 2020 and their symptom onset date from the official website of the Seoul Metropolitan Government and the district office, we calculated R_t_ using program R’s package “EpiEstim”. For asymptomatic cases, their symptom onset date was considered as -2, -1, 0, +1, and +2 days of confirmed date.

**RESULTS:**

Based on the information of 313 confirmed cases, the epidemic curve was shaped like ‘propagated epidemic curve’. The daily R_t_ based on R_t_c_ peaked to 2.6 on February 20, 2020, then showed decreased trend and became <1.0 from March 3, 2020. Comparing both R_t_ from R_t_c_ and from the number of daily onset cases, we found that the pattern of changes was similar, although the variation of R_t_ was greater when using R_t_c_. When we changed assumed onset date for asymptotic cases (-2 days to +2 days of the confirmed date), the results were comparable.

**CONCLUSIONS:**

R_t_ can be estimated based on R_t_c_ which is available from daily report of the Korea Centers for Disease Control and Prevention. Estimation of R_t_ would be useful to continuously monitor the effectiveness of the quarantine policy at the city and province levels.

## INTRODUCTION

As of March 27, 2020, there were 504,806 confirmed cases of a coronavirus disease 2019 (COVID-19) reported worldwide and 134 countries were reporting community transmission [1]. In Korea, a total of 9,332 confirmed cases were reported from January 20, 2020, when the first case was confirmed, to March 27, 2020. The daily number of confirmed cases in Korea increased rapidly after a large-scale cluster of COVID-19 cases occurred in mid-February at the Sincheonji Church in Daegu, reaching 909 newly confirmed cases per day on February 29, 2020. After February 29, 2020 the number of new cases has decreased, but small and large outbreaks are still being reported nationwide, and the total number of new cases outside of Daegu has increased as the immigration of COVID-19 from foreign countries increases [1].

The reproduction number (R) is defined as the average number of infected people generated during the infectious period of an infected patient. It is an index to quantify the transmissibility of infectious disease. The R value varies over time during epidemic periods owing to various factors such as infection control strategies, pathogen characteristics, population immunity, and changes in contact behaviors between infectious and susceptible individuals. This time-varying R value is known as an instantaneous reproduction number or time-variant reproductive number (R_t_). Therefore, observing changes in R_t_ is an important indicator for evaluating the effectiveness of infection control strategies and monitoring the spread of infection [2]. This study estimated R_t_ of COVID-19 using the information on confirmed cases in Seoul, Korea. It also summarized the results using the existing R_t_ statistical software package, EpiEstim.

## METHODS

Daily numbers of confirmed cases were obtained from COVID-19 status reports provided by the official website of Seoul city [3]. Moreover, information such as the presence or absence of symptoms and time of symptom onsets in the confirmed cases was collected from the official websites of Seoul district offices. A total of 329 cases were confirmed as infected from January 23, 2020, when the first case was confirmed in Seoul, to March 22, 2020. Table 1 shows the basic characteristics of these confirmed cases.

Software packages such as R_0_ and EpiEstim that are optimized for estimating R_t_ have been developed [4,5]. In this study, EpiEstim was used because it was developed recently and it requires less computing time than the other [5]. Generation time, which is required to calculate R_t_, is a time interval between infector’s infected date and its consecutive infectee’s infected date. However, generation time is usually estimated based on the difference between the time of symptom onset of the infector and the infectee, which is called serial interval, as it is often difficult to know the exact time of infection [6]. This study assumed the serial interval as gamma distributed with a mean of 3.96 days and a standard deviation of 4.75 days, which was reported in China [7].

R_t_ was estimated based on the daily number of confirmed cases (Rt_c) and symptom onset (Rt_s) because it is difficult to identify the exact time of infection. Twelve confirmed cases before February 16, 2020 were excluded from the analysis because from January 23, 2020 to February 16, 2020, there was no Rt_c for a considerable period, thus we could not assume that they were infected from previous case within Seoul. Four more confirmed cases were excluded with symptoms but their onset dates of symptoms were missing. Finally, 313 confirmed cases were included in the analysis. In asymptomatic cases, the time of symptom onset was assumed to be the same as the time of diagnosis (n= 70). In sensitivity analysis, R_t_ was calculated by assuming the times of symptom onset in asymptomatic cases as confirmed date (T_D_)-2 days, T_D_-1 day, T_D_+1 day, and T_D_+2 days. Then, those calculated R_t_ were compared to the calculated R_t_ with assumption of asymptomatic cases’ symptom onset date as T_D_. The data analysis was performed using the R version 3.6.3 (https://cran.r-project.org/bin/windows/base/old/3.6.3/) and EpiEstim, and the median R_t_ and 95% confidence intervals (CIs) were obtained.

### Ethics statement

This study uses data from official websites which are opened to public. So, this study is subject to institutional review board exception in accordance with article 13 of the enforcement ordinance (study using existing data or documents on subjects, etc.).

## RESULTS

**Figure 1.**
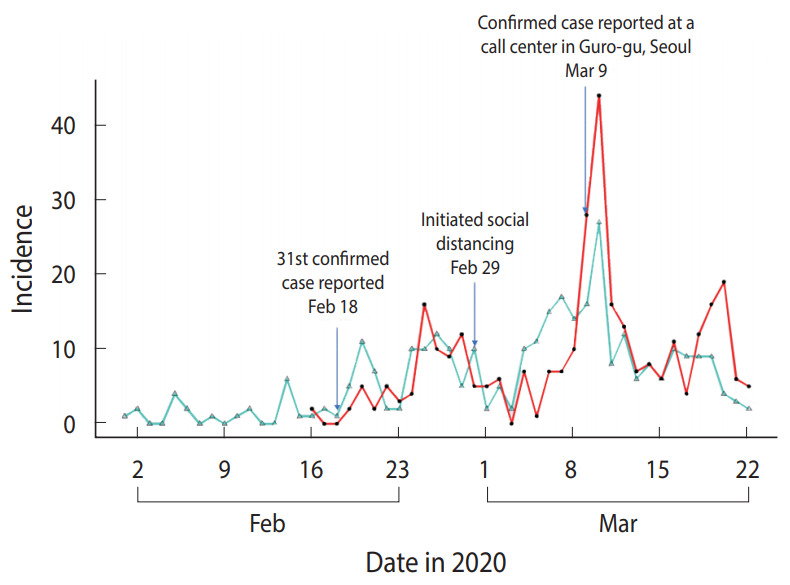
Distribution of the number of daily confirmed cases and symptom onsets in 313 confirmed coronavirus disease 2019 (COVID-19) cases in Seoul, Korea. The red line indicates the confirmed date, and the turquoise line indicates the onset date.

**Figure 2.**
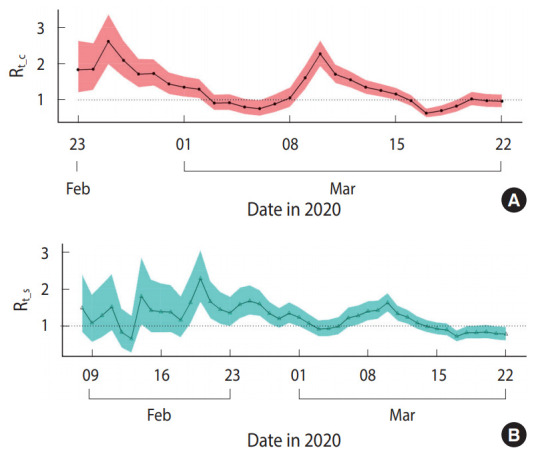
Reproductive number (R_t_) with 95% percentile confidence intervals in Seoul from February 23, 2020 to March 22, 2020. (A) Based on the daily number of confirmed cases (R_t_c_). (B) Based on the daily number of symptom onsets (R_t_s_). The R_t_s_ begins on February 8, 2020.

Figure 1 shows the distribution of the Rt_c and Rt_s in 313 confirmed cases included in the analysis. Figure 2A presents the median and 95% CI of R_t_ estimated using T_D_ information. Assuming this R_t_ as R_t_c_, R_t_c_ exhibited a decreasing trend from February 25, 2020 to March 6, 2020, which fell below 1 and then increased. It decreased again on March 10, 2020 and has shown a value of ≤ 1 after March 16, 2020. The median value and 95% CI of R_t_, which was estimated using the R_t_s_, are shown in Figure 2B. Assuming this R_t_ as R_t_s_, R_t_s_ decreased to < 1 from February 20, 2020 to March 4, 2020, it increased shortly, and then decreased again from March 10, 2020 remaining below 1 after March 14, 2020.

**Figure 3.**
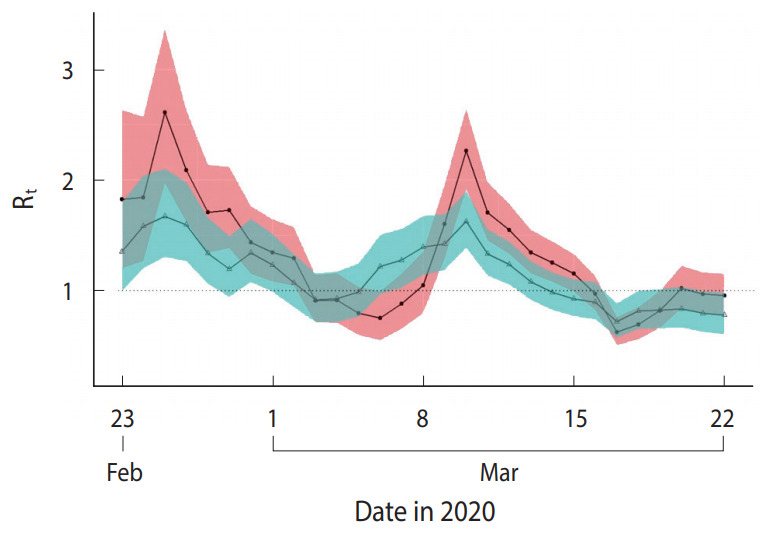
Comparison of the reproductive number (R_t_) between the results based on daily number of confirmed cases (R_t_c_) and results based on daily number of symptom onset (R_t_s_) in Seoul, Korea from February 23, 2020 March 22, 2020.

Figure 3 compares the mean R_t_ calculated based on the R_t_c_ and R_t_s_. Overall, R_t_c_ and R_t_s_ showed similar changes in pattern; however, R_t_c_ showed more abrupt changes than R_t_s_, and its highest and lowest values were estimated to be higher or lower than R_t_s_, respectively. Moreover, the points in time when the uptrend or downtrend changes occurred were approximately 1 day after R_t_s_, reflecting the lag time for testing and to confirm the diagnosis after symptom onset.

In the primary analysis, the symptom onsets of asymptomatic patients (n= 70) were assumed to be the same as T_D_. To perform sensitivity analysis, R_t_ were calculated and compared, assuming the times of symptom onset were the same as T_D_-2 days, T_D_-1 day, T_D_+1 day, and T_D_+2 days, relative to T_D_. Under each assumption, the 95% CI of the R_t_ values overlapped considerably, and the changes over time showed a similar pattern, as shown in the Supplementary Material 1.

## DISCUSSION

In this study, the daily R_t_ of COVID-19 in Seoul from February 23, 2020 to March 22, 2020, was estimated using the data on the Rt_c and Rt_s. Daily R_t_ refers to the infectivity of newly confirmed cases on day t. In other words, an increase in R_t_ indicates that infected individuals are likely to transmit the disease more actively than previously, and this requires more intensive interventions for infection control. In contrast, current strategies to control infection are effective when R_t_ decreases. If the R_t_ constantly remains below 1, the epidemic will be disappeared [3].

When the R_t_ was estimated using the Rt_c and Rt_s in Seoul, R_t_ values decreased from late February to early March and remained below 1 with some variations. This is possibly owing to the raised awareness of the public, enhanced infection control strategies, and social distancing, following a cluster outbreak that occurred at the Shincheonji Church in Daegu, Korea. The number of confirmed cases increased rapidly after another cluster outbreak was reported on March 10, 2020 at a call center in Guro-gu, Seoul. The R_t_ values remained higher than 1, reflecting the high transmission of the disease. However, the R_t_ decreased without further increase and has remained below 1 since March 10, 2020, after implementing infection control guidelines in high-risk workplaces, recommending the prohibition of mass gatherings, and limiting religious and public facility use as well as enhanced social distancing.

The R_t_ values obtained using the Rt_c and R_t_s_ were comparable, despite of slight differences. When the Rt_c was used in the calculation, variations in the R_t_ were greater as the total number of confirmed cases increased rapidly. This was due to an increase in diagnostic testing after more investigations were completed following outbreaks or policy implementations. However, the variations in R_t_ declined when the values were calculated using the daily number of symptom onsets because symptom onsets are distributed widely before and after T_D_. Nevertheless, trends in R_t_ value changes were similar in both cases. In COVID-19, most patients developed mild initial symptoms, which made it difficult to determine the exact time of symptom onset. Therefore, the similar pattern of the estimated R_t_ indicate that the R_t_ using the R_t_c_ may be useful to estimate the pattern of infection transmission and to evaluate the effectiveness of the infection control strategies in the early stages.

In the sensitivity analysis conducted in the asymptomatic cases with an assumption that the time of symptom onset was the same as T_D_-2 days to T_D_+2 days, the trend in estimated R_t_ showed similar changes in original data. The original data assumed that asymptomatic cases’ symptom onset dates were same as T_D_. Accordingly, the estimated R_t_ fells within the 95% CI of the original data. This indicates that assuming the time of symptom onset in asymptomatic cases as same as the date of confirmation is not significantly different from other assumptions in asymptomatic cases.

There are several points to consider when interpreting R_t_ obtained in our study. First, the R_t_ may be underestimated as it gets closer to the latest date. The reason is that fewer values of symptom onset are used for the R_t_ value estimation than the actual number of confirmed cases due to lag time between symptom onset and confirmed date. Second, our study used Chinese serial interval as the generation time to estimate R_t_. Since the generation time may be different from China, it is necessary to estimate R_t_ based on the estimated generation time in Korea, especially in Seoul. Third, the foreign immigration of confirmed cases was not considered. More accurate R_t_ prediction is possible if the infection transmission is traced accurately and confirmed whether there was an immigration of confirmed cases form outside of Seoul.

In conclusion, the R_t_ estimated from using the Rt_c and Rt_s in Seoul were both useful in evaluating effectiveness of the infection control strategies. The values have remained below 1 since March 15, 2020, indicating a decreased rate of infection transmission from confirmed cases in the community. However, further studies should be conducted as the influx of confirmed cases from abroad and from other regions in Korea are increasing. Furthermore, the effectiveness of the infection control strategies should be monitored constantly at local and national levels, using R_t_ estimated with the Rt_c and Rt_s.

## Figures and Tables

**Table 1.** Baseline characteristics of 329 confirmed coronavirus disease 2019 (COVID-19) case in Seoul from January 23, 2020 to March 22, 2020

Characteristics	n (%)
Sex	
Male	161 (48.9)
Female	168 (51.1)
Age (yr)	
<10	5 (1.5)
10-19	11 (3.3)
20-29	83 (25.2)
30-39	51 (15.5)
40-49	55 (16.7)
50-59	70 (21.3)
60-69	31 (9.4)
70-79	15 (4.6)
80-89	6 (1.8)
≥90	2 (0.6)
Days since symptoms to diagnosis (d)^1^	
Asymptomatic	70 (21.5)
≤ 3	113 (34.8)
4-7	85 (26.1)
> 7	57 (17.5)

1 Those who do not have symptom onset information (n=4) are not calculated.

## SUPPLEMENTARY MATERIALS

Korean version is available at http://www.e-epih.org/.
