# Supplementary material for: Time-variant reproductive number of COVID-19 in Seoul, Korea
Source: Epidemiol Health. 2020 Jun 28;42:e2020047. doi: 10.4178/epih.e2020047 (PMC7644928; doi:10.4178/epih.e2020047)
Supplement: Supplementary file 1 [file epih-42-e2020047-suppl1.pdf]

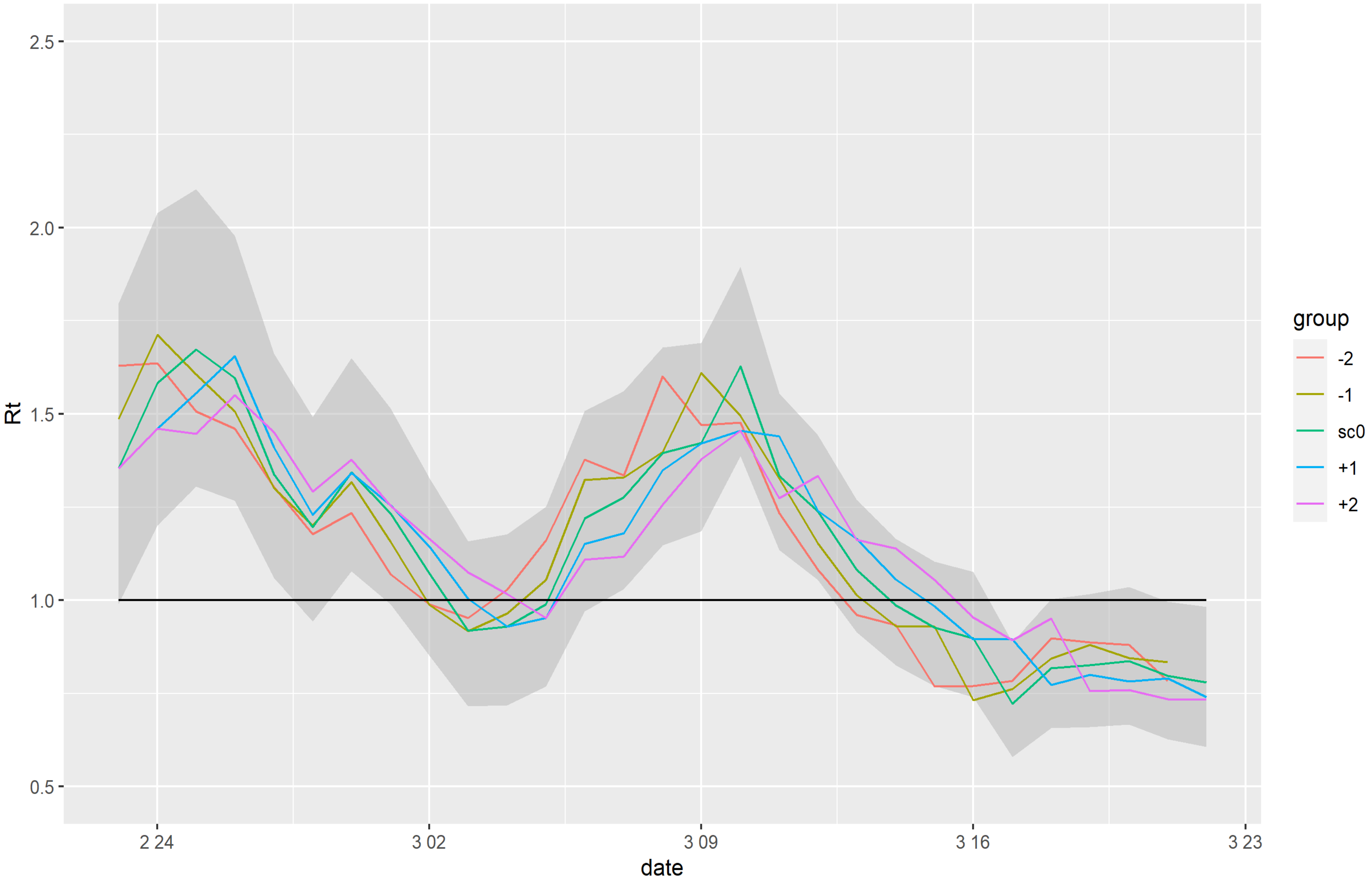

Supplementary Material 1. Sensitive analysis for assuming asymptomatic confirmed cases' symptom onset as "confirm date"('sc0' in this figure). Other assumption are shown in this figure as 2 days before confirm ('-2'), 1 day before confirm ('-1'), 1 day after confirm ('+1'), and 2 days after confirm ('+2').
