# Supplementary material for: Time-variant reproductive number of COVID-19 in Seoul, Korea
Source: Epidemiol Health. 2020 Jun 28;42:e2020047. doi: 10.4178/epih.e2020047 (PMC7644928; doi:10.4178/epih.e2020047)
Supplement: Supplementary file 2 [file epih-42-e2020047-suppl2.docx]

## **Title**: Time variant reproductive number of COVID-19 in Seoul, South Korea

Seong-Geun Moon^1^, Yeon-Kyung Kim^2^, Woo-Sik Son^3^, Jong-Hoon Kim^4^ , Jungsoon Choi^1^, Baeg-Ju Na^5^, Boyoung Park^1^

1) Hanyang University

2) Seoul Center for Infectious Disease Control and Prevention

3) National Institute for Mathematical Sciences

4) International Vaccine Institute

5) Seoul Metropolitan Government Civil Health Bureau

## **ORCID**

Seong-Geun Moon 0000-0002-5019-3483

Yeon-Kyung Kim 0000-0002-8417-0730

Woo-Sik Son 0000-0002-0421-4546

Jong-Hoon Kim 0000-0002-9717-4044

Jungsoon Choi 0000-0001-6815-1006

Baeg-Ju Na 0000-0002-3742-6035

Boyoung Park 0000-0003-1902-3184

## **Funding:** This research was supported by Government-wide R&D Fund project for infectious disease research (GFID), Republic of Korea (grant number: HG18C0088).

## **Correspondence to**

Boyoung Park, Department of Preventive Medicine, Hanyang University, 222, Wangsimni-ro, Seongdong-gu, Seoul, Republic of Korea

E-mail: hayejine@hanmail.net

## **Abstract**

Objectives: 서울시 Coronavirus disease 2019 (이하 COVID-19) 일별 확진자 수 및 확진자의 증상 발현일 정보를 기존 개발된 실시간감염재생산수(R_t_) 추정 통계 패키지에 적용하고, 이를 이용해 방역정책의 효과를 모니터링 할 수 있도록 한다.

Methods: 2020년 1월 23일부터 3월 22일까지 서울시 확진자 329명 중 서울시 및 구청의 공식 홈페이지에서 확진자 관련 정보가 확인되는 313명의 확진일과 증상 발현일 자료를 가지고 프로그램 R의 패키지 ‘EpiEstim’을 이용하여 R_t_를 산출하였다. 무증상 확진자의 증상 발현일 추정과 관련해서 민감도 분석을 함께 시행하였다.

Results: 분석에 사용된 313명의 확진자 정보를 토대로 그린 유행 곡선은 ‘접촉감염으로 인한 증식형 유행곡선’ 모양임을 확인하였다. 같은 대상에서 일별 확진자 수와 일별 증상 발현자 수를 이용하여 R_t_ 를 추정하였을 때 전반적으로 그 값이 2월 말부터 감소하여 3월 3일에는 1 미만으로 감소하였다. 일별 확진자 수와 일별 증상 발현자 수를 이용하여 구한 R_t_는 확진자 수를 이용할 경우 값이 더 크게 변화하는 차이가 있으나 변화 양상은 유사함을 확인하였다. 무증상 확진자의 증상 발현일을 확진일 전후로 가정하더라도 증상 발현일을 확진일로 가정했을 때와 결과가 유사함을 확인하였다.

Conclusion: 우리나라 전체와 각 시도별 일별 증상 발현자 수 또는 확진자 수를 이용하여 추정한 R_t_로 방역정책의 효과를 국가 및 시도 수준에서 지속적으로 모니터링 할 필요가 있다.

## **Keyword:** time variant reproductive number, Rt

# **Introduction**

2020년 3월 27일 기준 전 세계적으로 504,806명의 신종코로나감염증-19 (COVID-19) 확진자가 보고되었으며, 134개 국가에서 지역 감염이 일어나고 있다 (1). 한국에서는 2020년 1월 20일 첫 확진자가 발견된 이후 2020년 3월 27일까지 총 9,332명의 확진자가 보고되었다. 한국의 일별 확진자 수는 2월 중순 경 대구 신천지 교회에서 대규모 집단 감염이 발생한 이후 빠르게 증가하여 2월 29일 하루 909명의 신규 확진자가 발생한 이후 감소하고 있다. 그러나 각 중소규모 집단 발병들이 전국 곳곳에서 발생하고 있고, 해외 유입 확진자가 증가하면서 대구 외 지역의 확진자들은 증가하고 있는 추세를 보이고 있다 (1).

감염재생산수(R)는 집단 내에서 감염성이 있는 환자가 감염 가능 기간에 직접 감염시키는 평균 인원수로 정의하며, 감염병이 확대되어 나아가는 전파력을 정량화하는 지표이다. 감염재생산수는 방역 정책, 병원체 특성, 인구집단의 면역력, 감염원-감수성자 접촉 등 다양한 요인에 의해 유행 기간 중 시간에 따라 변하는 모습을 보인다. 이렇게 시간에 따라 변화하는 R을 실시간감염재생산수(time variant reproductive number, instantaneous reproduction number, R_t_)라 하며 R_t_의 변화를 보는 것은 방역정책의 효과 평가 및 감염 전파 양상을 감시하는데 중요한 지표가 된다 (2).

본 연구는 서울시의 확진자 정보를 이용하여 실시간감염재생산수(R_t_)를 추정하고, 기존 개발된 R_t_ 산출 통계 패키지(EpiEstim)를 통계 비전문가도 쉽게 적용할 수 있게 정리하였다.

# **Methods**

서울시의 COVID-19 발생 패턴을 확인하기 위해 서울시 코로나19 현황 공식 홈페이지를 이용하여 일별 확진자 수를 획득하였다 (3). 또한 서울시 및 구청 공식 홈페이지에서 확진자의 증상 여부, 증상 발현일 정보를 수집하였다. 이를 통해 서울시에서 처음 확진자가 발생한 2020년 1월 23일부터 3월 22일까지 확진된 총 329명을 확인하였다. 이들의 기본 특성을 Table 1에 제시하였다.

R_t_를 추정하는데 최적화된 R0와 EpiEstim 등의 패키지가 개발되어 있는데 (4, 5), 본 연구에서는 상대적으로 최근에 개발되었고, 모델이 간단하여 시간과 컴퓨팅 비용이 적게 드는 EpiEstim을 적용하였다 (5). R_t_를 계산하기 위해 필요한 정보인 세대기 (generation time)는 감염원 (infector)이 감염자 (infectee)를 감염시키는데 까지 걸리는 시간을 이용하여 추정하며, 이를 세대 간격 (generation interval), 연속세대기간 (serial interval)이라 부르기도 한다. 그러나 정확한 감염 일자를 확인하기 어려운 경우가 많기 때문에 일반적으로는 감염원 (infector)과 감염자 (infectee)의 증상 발현일의 차이로 세대기를 추정한다 (6). 본 연구에서는 중국에서 발표한 연속세대기간을 이용하여, 평균 3.96일, 표준편차 4.75일 (7)의 감마 분포를 따른다고 가정하였다.

R_t_를 산출하기 위해서는 감염일을 사용하는 것이 이상적이나, 정확한 감염 시점을 알기 어렵기 때문에, 일 확진자 수와 일별 증상 발현자 수를 기반으로 R_t_를 추정하였다. 상당 기간 동안 일별 확진자 수가 0건이어서 이전 확진자가 다른 사람을 감염시켰다고 보기 어려운 기간을 제외하기 위해 2월 16일 이전 확진자 12명은 분석에서 제외하였고, 증상이 있다고 응답하였으나 증상 발현일 정보를 알 수 없는 4명을 추가적으로 제외하여 최종적으로 313명을 분석에 포함하였다. 무증상 확진자의 경우, 증상 발현일은 확진일과 동일하다고 가정하였다 (n=70). 이 가정이 결과의 신뢰도에 영향 미치지 않을지를 확인해보기 위해 무증상 확진자의 증상 발현일을 확진일 2일 전, 1일 전, 1일 이후, 2일 이후로 가정하여 R_t_를 산출하였고, 이를 원래의 추정치와 비교하는 민감도 분석을 시행하였다. 프로그램 ’R’ ver. 3.6.3과 EpiEstim 패키지를 사용하여 분석을 진행하였으며, R_t_의 중앙값과 95% 신뢰구간을 제시하였다.

# **Results**

분석에 포함된 313명의 증상 발현일 분포와 확진일 분포는 Figure 1과 같다. 확진일 정보를 이용하여 추정한 R_t_의 중앙값 및 신뢰구간은 Figure 2(A)와 같다. 이 R_t_를 R_t__c라 하면, R_t__c는 2월 25일부터 3월 6일까지 감소하는 추세를 보여 1 미만이 되었다가 그 이후 증가하였다 3월 10일 이후 감소하여 3월16일 이후로는 1 또는 그보다 낮은 값을 보이고 있다. 증상 발현일 정보를 이용하여 추정한 R_t_의 중앙값 및 신뢰구간은 Figure 2(B)에 제시되어 있다. 이를 R_t__s라 하면, R_t__s는 2월 20일부터 3월 4일까지 감소하여 1미만이 되었다가 이후 상승하고 3월 10일부터 감소하여 3월 14일 이후로는 1 미만의 값을 보이고 있다.

Figure 3은 확진일(R_t__c)과 증상 발현일(R_t__s)로 각각 계산한 R_t_의 중앙값을 비교하고 있다. 전반적으로 R_t__c와 R_t__s는 유사한 변화 양상을 보이고 있으나 R_t__c는 R_t__s보다 급격한 변화양상을 보이고 있으며, 그 최고점 및 최저점도 R_t__s보다 더 높거나 낮게 추정되고 있다. 또한 증가/감소의 추이가 변화하는 시점은 R_t__s보다 약 1일 늦게 나타나고 있는데 이는 증상 발현 이후 검사 및 확진 판정을 받는데 걸리는 시간이 반영된 결과로 보인다.

기본 분석에서는 무증상 확진자들(n=70)의 증상 발현일이 확진일과 같다고 가정하였는데 이에 대한 민감도 분석을 위해 증상 발현일을 확진일 대비 2일 전, 1일 전, 1일 이후, 2일 후와 같다 가정하고 Rt를 산출하였 비교하였다. 각각의 가정하에서 R_t_값의 95% 신뢰구간이 상당 부분 겹치는 것으로 나타났으며 시간에 따른 패턴 역시 비슷한 양상을 보였다. 이는 supplementary figure로 제시하였다

# **Discussion**

본 연구에서는 서울시의 일별 확진자 수 및 증상 발현일 자료를 이용하여 2020년 2월 23일에서 3월 22일까지의 서울시의 일별 실시간감염재생산수를 추정하였다. 일별 R_t_는 t일에 전체 감염자가 갖는 감염성 대비 t일에 새로 감염된 감염자가 갖는 감염성을 의미한다. 즉, R_t_값이 증가하는 경향이라면 이는 현재 감염된 사람들이 다른 사람들을 전보다 더 활발하게 더 감염시킬 것이라는 의미이며 더욱 강력한 방역 대책 또는 추가적인 방역대책이 필요함을 의미한다. 반대로 R_t_값이 감소한다면 이는 방역대책의 효과가 있다고 볼 수 있으며, R_t_ 값이 지속적으로 1이하의 값을 보이면 질병의 유행이 사라지게 된다 (3).

서울시에서 일별 확진자 수와 일별 증상 발현자 수를 이용하여 R_t_ 를 추정하였을 때 약간의 차이는 있으나 2월 말부터 3월 초까지 R_t_값이 감소하는 추세를 보여 1 미만으로 감소하였다. 이는 대구에서 발생한 신천지 집단감염과 이에 따른 COVID-19에 대한 사람들의 위기 의식 및 강화된 방역 대책, 사회적거리두기 운동 등의 효과로 인한 것으로 추정된다. 이후 구로구 콜센터 클러스터의 발생으로 3월 10일 근처 확진자 수가 급증하고, 이를 반영하듯 Rt가 1 이상 높게 측정되었다. 하지만 3월 10일 이후 추가된 고위험사업장 관리지침, 종교시설 및 단체시설 사용 및 집회 금지 권고, 강화된 사회적 거리두기 운동 등의 효과로 클러스터 조사 후 R_t_가 추가적 상승 없이 다시 감소하여 1 미만으로 유지되는 모습을 보였다.

일별 확진자 수와 일별 증상 발현자 수를 이용하여 구한 R_t_는 그 값에는 다소 차이가 있었다. 다만 일별 확진자 수를 쓰는 경우, 특정 사건이나 정책 등에 따라 전수조사 등으로 검사 건수가 증가하고, 이에 따라 확진자 수가 급증하여 Rt 값의 변동이 커지는 경향을 보인다. 그러나 확진자의 증상 발현일은 확진일 전후로 다양하게 분포하게 되므로 일별 증상 발현자 수를 이용하여 R_t_를 산출했을 때 그 변동은 일별 확진자 수에 기반한 결과보다 변동이 작아진다. 그러나 Rt의 변화 추이는 유사하였다. COVID-19의 경우 대부분의 경우 초기 증상이 경미하여 정확한 증상 발현일을 추정하기가 어려운 점을 고려하였을 때 일별 확진자 수 정보만을 이용하여 추정한 R_t_가 증상 발현일을 이용한 결과와 유사한 변화 양샹을 보이는 것은 일별 확진자 수를 이용하여 추정한 R_t_ 도 방역정책의 효과 평가를 조기에 시행하며 감염 전파 양상을 추정하는 데에 효과적으로 이용할 수 있다는 것을 의미한다.

무증상 확진자의 증상 발현일은 확진일과 동일하다 가정하고 R_t_를 추정했다는 점에 대해 실시한 민감도 분석에서는 증상 발현일을 조절하여도 R_t_의 증감 추세는 확진일과 동일하다고 가정한 원래 결과와 유사한 변화를 보이며, 그 값이 원래 결과의 95% 신뢰구간 안에 있는 모습을 확인하였다. 이는 무증상 확진자의 증상 발현일을 확진일로 가정하는 것이 다른 가정과 비교하여 결과 차이가 크지 않음을 의미한다.

우리의 연구에서 R_t_ 를 해석하는데 있어 몇가지 고려해야 될 사항들이 있다.

첫째, 증상 발현일을 변수로 사용할 경우, 최근 증상이 발현된 사람은 검사를 받고 결과 확인까지 시간이 지난 뒤에야 확진 여부를 알 수 있다. 따라서 최근 날짜로 다가올수록 실제 확진자 수 보다 적은 수의 확진자의 증상 발현일이 R_t_추정에 사용되어 실제 R_t_ 를 과소추정할 수 있다. 둘째, 중국에서 추정한 세대기 및 그 분포는 한국과 다를 수 있기 때문에 추후 국내 특히 서울지역의 세대기를 추정하여 그에 기반한 R_t_를 추정할 필요가 있다. 셋째, 유입 환자에 대한 고려가 이루어 지지 못하였다. 추후 감염경로가 뚜렷하게 밝혀져 서울 외부에서 감염자가 유입된 것인지 여부를 확인할 수 있다면 좀 더 정확한 R_t_ 예측이 가능할 것으로 보인다.

결론적으로 서울시의 일별 확진자 수 및 증상 발현자 수 자료를 이용하여 추정한 R_t_는 두 자료 모두 방역정책의 효과를 추정하는 데에 용이했으며, 3월 15일 이후에는 1 미만으로 유지되는 모습을 보여, 확진자의 지역사회 내 감염률은 감소했다고 할 수 있다. 그러나 향후 연구에서는 증가하는 해외 유입 확진자와 타지역 확진자들의 감염도 고려해야 할 것이며, 더 나아가 우리나라 전체와 각 지역별 일별 증상 발현자 수 또는 확진자 수를 이용하여 추정한 R_t_로 방역정책의 효과를 국가 및 지역 수준에서 지속적으로 모니터링 할 필요가 있다.

## **Disclosure**

All authors have no potential conflicts of interest to disclose.

## **Acknowledgments**

We thanks to all of the persons who were struggling in healthcare fields to overcome the COVID19 outbreak. This study were performed under the research project named ‘Research and Development on Integrated Surveillance System for Early Warning of Infectious Diseases (RISEWIDs)’. The investigators of this project were Jaiyong Kim (Yonsei University), Sunworl Kim (National Medical Center), Eui Jung Kwon (National Health Insurance Review and Assessment Service), Dong Wook Kim (National Health Insurance Service), Moran Ki (National Cancer Center), Hyunjin Son (Pusan National University Hospital), Jong-Hun Kim (Sungkyunkwan University), Jin Yong Kim(Incheon Medical Center), Heeyoung Lee (Seoul National University Bundang Hospital), Boyoung Park (Hanyang University), Woo-Sik Son (National Institute for Mathematical Sciences), Jungsoon Choi (Hanyang University), Sunhwa Choi (National Cancer Center), Okyu Kwon (National Institute for Mathematical Sciences), Hyojung Lee (National Institute for Mathematical Sciences), Jong-Hoon Kim (International Vaccine Institute), Heecheon Kim (MISO INFO TECH), and Bo Youl Choi (Hanyang University). This project was supported by Government-wide R&D Fund Project for Infectious Disease Research (GFID), Republic of Korea (Grant No. HG18C0088).

# **Reference**

1. Korean Centers for Disease Control and Prevention [cited 2020 March 27]. Available from: http://ncov.mohw.go.kr/.

2. Lipsitch M, Bergstrom CT. Invited commentary: real-time tracking of control measures for emerging infections. American journal of epidemiology. 2004;160(6):517-9; discussion 20.

3. Webpage for Seoul [cited 2020 March 27]. Available from: http://www.seoul.go.kr/coronaV/coronaStatus.do/.

4. Obadia T, Haneef R, Boelle PY. The R0 package: a toolbox to estimate reproduction numbers for epidemic outbreaks. BMC medical informatics and decision making. 2012;12:147.

5. Cori A, Ferguson NM, Fraser C, Cauchemez S. A new framework and software to estimate time-varying reproduction numbers during epidemics. American journal of epidemiology. 2013;178(9):1505-12.

6. Vink MA, Bootsma MCJ, Wallinga J. Serial Intervals of Respiratory Infectious Diseases: A Systematic Review and Analysis. American journal of epidemiology. 2014;180(9):865-75.

7. Du Z, Xu X, Wu Y, et al. Serial Interval of COVID-19 among Publicly Reported Confirmed Cases. Emerging Infectious Diseases. 2020;26(6):1341-1343.

**Figure legends**

## **Figure 1.** Distribution of number of daily confirmed cases and onset of symptoms in 313 confirmed COVID-19 case in Seoul. Red line for confirmed date, Turquoise line for onset date.

## **Figure 2.** R_t_ with 95% percentile confidence intervals in Seoul from Feb 23, 2020 to March 22, 2020. Upper figure is based on daily number of confirmed cases(R_t__c). Lower figure is based on daily number of symptom onset(R_t__s). R_t__s begins at Feb 8, 2020.

## **Figure 3.** Comparison of Rt between the result based on daily number of confirmed cases(R_t__c) and result based on daily number of symptom onset(R_t__s) in Seoul from Feb 23, 2020 to March 22, 2020

**Table 1.** Baseline characteristics of 329 confirmed COVID-19 case in Seoul from Jan 23, 2020 to Mar 22, 2020

|  | N (%) |
| --- | --- |
| Sex |  |
| Male  Female | 161 (48.9%)  168 (51.1%) |
| Age |  |
| <10  10-19  20-29  30-39  40-49  50-59  60-69  70-79  80-89  90 ≥ | 5 (1.5%)  11 (3.3%)  83 (25.2%)  51 (15.5%)  55 (16.7%)  70 (21.3%)  31 (9.4%)  15 (4.6%)  6 (1.8%)  2 (0.6%) |
| Days since symptom to diagnosis* | |
| Asymptomatic ≤ 3 days 4-7 days > 7 days | 70 (21.54%)  113 (34.77%)  85 (26.15%)  57 (17.55%) |

* Those who don't have symptom onset information (n=4) are not calculated.
